# Supplementary material for: Ruling out pulmonary embolism across different healthcare settings: A systematic review and individual patient data meta-analysis
Source: PLoS Med. 2022 Jan 25;19(1):e1003905. doi: 10.1371/journal.pmed.1003905 (PMC8824365; doi:10.1371/journal.pmed.1003905)
Supplement: S1 Text — Includes a detailed statistical analyses plan (including references), Table A Diagnostic strategies under evaluation, Table B Data template, Table C Summary of missing data in each study, and Table D Summary of included studies. (DOCX) [file pmed.1003905.s002.docx]

**S1 Text.**

**Statistical analyses plan**

**Missing data**

In this large IPD dataset consisting of 23 studies, there were two types of missing values; partially or systematically missing. Partially missing values were missing in a certain proportion, but not completely in each study, while systematically missing values were completely missing in certain studies because they were not collected or could not be provided by the original study group. For example, quantitative D-dimer was completely observed in 3 studies (missing proportion = 0%), partially missing in 17 studies with the range of missing proportion within each study between 0.1% and 95.8%, and systematically missing (i.e. missing proportion = 100%) in 3 studies [1-3]. A simple solution for missing values is a complete case analysis (i.e. excluding patients who had at least one missing data from analyses), however, it has been reported to lead to less precise estimates when data are missing completely at random (MCAR) and even biased estimates when data are missing at random (MAR) [4]. Thus, multiple imputation is recommended to account for uncertainty caused by missing data [5]. In accordance with statistical recommendation, we planned to multiply impute missing values, as described in the protocol [6]. Recently, state-of-the-art statistical approaches have been reported to impute systematically missing values by using information of both within and between studies (multi-level imputation) [7-10]. However, we could not apply multi-level imputation because of non-convergence issues, likely caused by high proportion of systematically missing values. Therefore, we decided to impute only partially missing values within each study as we did in another IPD article for deep vein thrombosis [11]. Within each study, we applied multiple imputation technique with chained equations with all available information, including the outcome, except variables missing in more than 80% of patients in the study. Thus, we could not use 5 studies for the analyses in which quantitative D-dimer was necessary (3 studies with systematically missing quantitative D-dimer [1-3] and 2 studies with partially missing quantitative D-dimer in more than 80% of patients within each study [12,13]). Ten imputation datasets were created and the results of the analyses performed separately in each set were combined.

**Statistical analyses**

To evaluate the diagnostic performance of each strategy across different healthcare settings, we first estimated traditional diagnostic accuracy measures, i.e. sensitivity and specificity. Hereto, we constructed a multi-level (to account for the nesting of patients within studies) logistic model with the classification by each diagnostic strategy as binary outcome, i.e. the classification ‘further testing is necessary’ for those *with* PE, versus ‘PE can be excluded’ for those *without* PE, respectively [14]. Subsequently, disease status was added as a covariable to estimate sensitivity and specificity (i.e. for sensitivity ‘0’ for those without PE and ‘1’ for those with PE, and for specificity ‘0’ for those with PE and ‘1’ for those without PE). In addition, we added the interaction terms between ‘healthcare setting’ and the disease status variables. Due to non-convergence issues, we used univariate random effects modeling rather than bivariate modeling in accordance with methodological recommendation [15]. Next, for the analyses of failure rate, we constructed a multilevel logistic model with presence/absence of PE as outcome and classification by each diagnostic strategy as a covariable. Also, ‘healthcare setting’ and its interaction term with classification by each diagnostic strategy was added in the model. The failure rate of each diagnostic strategy can be quantified by estimating the probability of PE in patients categorized as ‘PE can be excluded’. This measure is frequently applied in the field of diagnostic studies in suspected PE [16]. Similarly, for the analyses of efficiency, a model with the classification by each strategy (i.e. ‘further testing is necessary’ versus ‘PE can be excluded’) as outcome and ‘healthcare setting’ as a covariable was used. Efficiency was then quantified as a probability of being classified as ‘PE can be excluded’ by each strategy. In both models for failure rate and efficiency, a random effect for the intercept was applied. All measures were estimated for each healthcare setting. For all accuracy measures – in addition to the 95% confidence intervals (CI) – 95% prediction intervals (PI) were estimated. 95% PI illustrates the performance that can be expected when the model is applied in a new population, taking heterogeneity across studies in account.

Forest plots were drawn to visualize the difference of each diagnostic strategy in terms of failure rate and efficiency. Finally, we assessed the overall effect of PE prevalence on failure rate and efficiency. Hereto, in accordance with a previous systematic review [17], log-transformed prevalence was added as continuous covariable to the models described above and the influence of prevalence of safety and efficiency on a continuous scale was plotted graphically.

**Table A: Diagnostic strategies under evaluation**

| **Diagnostic strategy** | Wells rule | | Revised Geneva rule | | YEARS algorithm | | PERC algorithm | | |
| --- | --- | --- | --- | --- | --- | --- | --- | --- | --- |
| **Items and points** | Previous VTE | 1.5 | Previous VTE | 3 | Clinical signs of DVT | | Age < 50 years | | |
|  | Heart rate > 100/min | 1.5 | Heart rate |  | Hemoptysis | | Heart rate < 100/min | | |
|  | Surgery or immobilization < 4 weeks | 1.5 | 75-94/min | 3 | PE most likely diagnosis | | Saturation > 94% | | |
|  | Hemoptysis | 1 | ≥ 95/min | 5 |  |  | No unilateral leg swelling | | |
|  | Active cancer | 1 | Surgery or fracture < 1 month | 2 |  |  | Hemoptysis | | |
|  | Clinical signs of DVT | 3 | Hemoptysis | 2 |  |  | No recent trauma or surgery | | |
|  | Alternative diagnosis less likely than PE | 3 | Active cancer | 2 |  |  | No estrogen use | | |
|  |  |  | Unilateral lower limb pain | 3 |  |  | No previous VTE | | |
|  |  |  | Pain on lower limb, deep venous palpation, and unilateral edema | 4 |  |  |  |  | |
|  |  |  | Age > 65 years | 1 |  |  |  |  | |
| **Pre-test probability assessment** | **The original classification** | | **The original classification** | | Low | 0 items | PE ruled out | | Fulfilling all the criteria above |
|  | Low | 0-1.5 | Low | 0-3 | High | ≥ 1 items |  | |  |
|  | Intermediate | 2-6 | Intermediate | 4-10 |  |  |  | |  |
|  | High | ≥ 6.5 | High | ≥11 |  |  |  | |  |
|  | Unlikely | 0-4 | **For only D-dimer adjusted to pre-test probability,** | |  |  |  | |  |
|  | Likely | ≥ 4.5 | Low | 0-5 |  |  |  | |  |
|  | **For only D-dimer adjusted to pre-test probability,** | | Intermediate | 6-10 |  |  |  | |  |
|  | Low | 0-4 | High | ≥11 |  |  |  | |  |
|  | Moderate | 4.5-6 |  |  |  |  |  | |  |
|  | High | ≥ 6.5 |  |  |  |  |  | |  |
| **Assessment of D-dimer testing** |  |  |  |  |  |  |  |  | |
| Qualitative D-dimer | Normal | Negative | Normal | Negative |  |  |  |  | |
|  | Abnormal | Positive | Abnormal | Positive |  | NA |  | NA | |
| Quantitative D-dimer with the fixed cut-off | Normal | < 500 ng/ml | Normal | < 500 ng/ml |  |  |  |  | |
|  | Abnormal | ≥ 500 ng/ml | Abnormal | ≥ 500 ng/ml |  | NA |  | NA | |
| Quantitative D-dimer adjusted to age | Normal | < 500 ng/ml for younger than 50 years | Normal | < 500 ng/ml for younger than 50 years |  | NA |  | NA | |
|  |  | < Age * 10 for 50 years or older |  | < Age * 10 for 50 years or older |  |  |  |  | |
|  | Abnormal | ≥ 500 ng/ml for younger than 50 years | Abnormal | ≥ 500 ng/ml for younger than 50 years |  |  |  |  | |
|  |  | ≥ Age * 10 for 50 years or older |  | ≥ Age * 10 for 50 years or older |  |  |  |  | |
| Quantitative D-dimer adjusted to pre-test probability | Normal | < 1000 ng/ml for a low probability | Normal | < 1000 ng/ml for a low probability | Normal | < 1000 ng/ml for a low probability |  | NA | |
|  |  | < 500 ng/ml for a moderate probability |  | < 500 ng/ml for an intermediate probability |  | < 500 ng/ml for a high probability |  |  | |
|  | Abnormal | ≥ 1000 ng/ml for a low probability | Abnormal | ≥ 1000 ng/ml for a low probability | Abnormal | ≥ 1000 ng/ml for a low probability |  |  | |
|  |  | ≥ 500 ng/ml for a moderate probability |  | ≥ 500 ng/ml for an intermediate probability |  | ≥ 500 ng/ml for a high probability |  |  | |
| **Further testing** | For fixed or age-adjusted D-dimer testing, Unlikely, Low, or Intermediate plus Abnormal D-dimer testing, or High or Likely regardless of D-dimer testing. | | Low or Intermediate plus abnormal D-dimer testing or High regardless of D-dimer testing.  In all other patients, PE is considered ruled-out. | | Low or High plus abnormal D-dimer testing.  In all other patients, PE is considered ruled-out. | | All patients not fulfilling all the criteria above. In all other patients, PE is considered ruled-out. | | |
|  | For D-dimer adjusted to pre-test probability, Low or Moderate plus abnormal D-dimer testing, or high regardless of D-dimer testing | |  | |  | |  | | |
|  | In all other patients, PE is considered ruled-out. | |  |  |  |  |  | | |

Abbreviations: VTE, venous thromboembolism; DVT, deep vein thrombosis; PE, pulmonary embolism

**Table B: Data template**

| Variable | Label | Values |
| --- | --- | --- |
| Study | Study |  |
| Setting | Healthcare setting | 1: Self-referral emergency care, 2: Primary healthcare, 3: Referred secondary care, 4: Hospitalized or nursing home care |
| Inpatient | Inpatient/Outpatient | 0: Outpatient, 1: Inpatient |
| Age | In years |  |
| Sex | Male/female | 0: Male, 1: Female |
| Estrogen | Estrogen use | 0: No, 1: Yes |
| PreviousVTE | Previous venous thromboembolism  (per study definition) | 0: No, 1: Yes |
| ImmoSurg | Immobilization or suegery <4 weeks (Wells item) | 0: No, 1: Yes |
| Hemoptysis | Presence of hemoptysis | 0: No, 1: Yes |
| Cancer | Active cancer (per study definition) | 0: No, 1: Yes |
| SympDVT | Clinical signs of DVT (Wells item) | 0: No, 1: Yes |
| HR | Heart rate in bpm |  |
| Tachycardia | Heart rate > 100 | 0: No, 1: Yes |
| SpO2 peripheral | Oxygen saturation in % (pulse oximetry) |  |
| LegSwellingEdema | Unilateral leg swelling or edema | 0: No, 1: Yes |
| LegPain | Unilateral lower limb pain or deep venous palpation with unilateral edema | 0: No, 1: Yes |
| PEmostlikely | PE most likely diagnosis (Wells item) | 0: No, 1: Yes |
| DdimerQual | Qualitative D-dimer | 0: Negative, 1: Positive |
| DdimerQuant | Quantitative D-dimer in ng/ml |  |
| DdimerAssay | D-dimer assay | 0: Unknown, 1: VIDAS, 2: TinaQuant, 3: STA Liatest, 4: Innovance, 5: SimpliRED, 6: Simplify, 7: COBAS, 8: Others |
| VTEfinal | VTE yes or no | 0: No, 1: Yes |

**Table C: Proportion of missing data in each study**

|  | Sanson Thromb Haemost  2000 [3] | Wicki J Arch Intern Med 2001 [18] | Kline JA Ann Emerg Med 2002 [12] | Perrier A Am J Med 2004 [19] | Ghanima W J Thromb Haemost 2005 [20] | Perrier A N Engl J Med 2005 [21] | Kearon C Ann Intern Med 2006 [2] | Kline JA Chest 2006 [13] | van Belle A JAMA 2006 [22] | Goekoop RJ Thromb Haemost 2007 [23] | Runyon MS Emerg Med J 2007 [24] | Kline JA J Thromb Haemost 2008 [25] |
| --- | --- | --- | --- | --- | --- | --- | --- | --- | --- | --- | --- | --- |
| N | 517 | 1089 | 948 | 965 | 432 | 755 | 1123 | 2255 | 3296 | 876 | 1187 | 7889 |
| Age | 0 | 0 | 0 | 0 | 0 | 0 | 0 | 0 | 0 | 0 | 0 | 0 |
| Female sex | 0 | 0 | 0 | 0 | 0 | 0 | 0 | 0 | 0.3 | 0 | 0.1 | 0 |
| Inpatient | 0 | 0 | 0 | 0 | 0 | 0 | 0 | 0 | 0 | 0 | 0 | 0 |
| Previous VTE | 0.8 | 0 | 0 | 0.2 | 0.5 | 0 | 0 | 0 | 0.2 | 0.2 | 0.3 | 0 |
| Heart rate > 100/min | 1.2 | 0.1 | 0.4 | 0.4 | 4.2 | 0.4 | 0 | 1.2 | 0.2 | 0.1 | 53.2 | 0.3 |
| Surgery or immobilization < 4 weeks | 0.6 | 0 | 0 | 0 | 0 | 0 | 0 | 0.3 | 0.2 | 0 | 1.3 | 0 |
| Hemoptysis | 0.8 | 0 | 0.1 | 0 | 28.5 | 0 | 0 | 0 | 0.2 | 0.2 | 0.8 | 0 |
| Active cancer | 6.4 | 0 | 0.1 | 0.3 | 0.7 | 0 | 0 | 0 | 0.2 | 0.1 | 0.2 | 0 |
| Clinical signs of DVT | 0.2 | 37.3 | 0 | 0 | 28.7 | 0.1 | 0 | 0 | 0.2 | 0.1 | 1.3 | 0 |
| Alternative diagnosis less likely than PE | 0.4 | 100 | 100 | 3.5 | 100 | 1.1 | 0 | 20.1 | 0.2 | 0 | 1.3 | 0.1 |
| Qualitative D-dimer | 3.9 | 0 | 20 | 0 | 100 | 0.1 | 0.4 | 0 | 100 | 100 | 7 | 0.1 |
| Quantitative D-dimer | 100 | 0 | 88.9 | 0.1 | 0 | 1.2 | 100 | 95.8 | 15.6 | 8.9 | 5.1 | 64.5 |
| VTE | 0 | 0 | 0 | 0 | 0 | 0 | 0 | 0 | 0 | 0 | 0.2 | 0 |

|  | Righini M Lancet 2008 [26] | Douma RA Ann Itern Med 2011 [27] | Galipienzo J Rom J Intern Med 2012 [28] | Geersing GJ BMJ 2012 [1] | Kline JA J Thromb Haemost 2012 [29] | Mos ICM Thromb Res 2014 [30] | Righini M JAMA 2014 [31] | Schouten HJ J Am Geriatr Soc 2014 [32] | Penaloza A Lancet Haematol 2017 [33] | van der Hulle T Lancet 2017 [34] | Kearon C N Engl J Med 2019 [35] |
| --- | --- | --- | --- | --- | --- | --- | --- | --- | --- | --- | --- |
| N | 1692 | 807 | 240 | 597 | 678 | 279 | 3324 | 129 | 705 | 3448 | 2017 |
| Age | 0 | 0 | 0 | 0 | 0 | 0 | 0 | 0 | 0 | 0 | 0 |
| Female sex | 0 | 0 | 0 | 0 | 0 | 0.4 | 0 | 0 | 0 | 0 | 0 |
| Inpatient | 0 | 0 | 0 | 0 | 3.4 | 2.9 | 0 | 0 | 0 | 0 | 0 |
| Previous VTE | 0 | 0 | 0 | 0 | 0 | 0 | 0 | 1.6 | 0 | 0.1 | 0 |
| Tachycardia | 0.1 | 0 | 0 | 0 | 0 | 61.3 | 4.9 | 3.9 | 0 | 2 | 0 |
| Surgery or immobilization < 4 weeks | 0 | 0 | 0 | 0 | 0 | 24.4 | 0 | 0 | 0 | 0.1 | 0 |
| Hemoptysis | 0 | 0 | 0 | 0 | 0 | 26.2 | 0.1 | 1.6 | 0 | 0 | 0 |
| Active cancer | 0 | 0 | 0 | 0 | 0 | 20.8 | 0 | 0.8 | 0 | 0.1 | 0 |
| Clinical signs of DVT | 0.1 | 0 | 0 | 0 | 0 | 20.8 | 2.7 | 0 | 0 | 0 | 0 |
| Alternative diagnosis less likely than PE | 2.3 | 0 | 0 | 0 | 28.5 | 17.9 | 0 | 0 | 0 | 0 | 0 |
| Qualitative D-dimer | 0.5 | 100 | 100 | 0 | 100 | 100 | 100 | 7 | 100 | 100 | 100 |
| Quantitative D-dimer | 0.5 | 7.4 | 2.5 | 100 | 0 | 27.2 | 7.3 | 61.2 | 13.2 | 0.2 | 0.6 |
| VTE | 0 | 0 | 0 | 0 | 0 | 0 | 0 | 0.8 | 0 | 0 | 0 |

Abbreviations: N, number of patients; VTE, venous thromboembolism; DVT, deep vein thrombosis; PE, pulmonary embolism

**Table D: Summary of included studies**

| **Author** | **Journal** | **Publication year** | **Setting** | **Number of patients^a^** | **Number of PE patients^a^** | **Prevalence (%)^a^** | **D-dimer assay** |
| --- | --- | --- | --- | --- | --- | --- | --- |
| Sanson BJ [3] | Thromb Haemost | 2000 | Referred secondary care and inpatients | 517 | 160 | 30.9 | qualitative (NR) |
| Wicki J [18] | Arch Intern Med | 2001 | Referred secondary care | 1089 | 296 | 27.2 | quantitative (Asserachrom D-Di enzyme / VIDAS (bioMérieux)) |
| Kline JA [12] | Ann Emerg Med | 2002 | Self-referral emergency care | 948 | 186 | 19.6 | qualitative (NR) |
| Perrier A [19] | Am J Med | 2004 | Referred secondary care | 965 | 229 | 23.7 | quantitative  (VIDAS (bioMérieux)) |
| Ghanima W [20] | J Thromb Haemost | 2005 | Referred secondary care | 432 | 95 | 22.0 | quantitative (STA Liatest) |
| Perrier A [21] | N Engl J Med | 2005 | Referred secondary care | 755 | 197 | 26.1 | quantitative (VIDAS (bioMérieux)) |
| Kearon C [2] | Ann Intern Med | 2006 | Primary healthcare and inpatients | 1123 | 168 | 15.0 | qualitative (SimpliRED) |
| Kline JA [13] | Chest | 2006 | Self-referral emergency care | 2255 | 108 | 4.8 | qualitative (Simplify) |
| van Belle A [22] | JAMA | 2006 | Referred secondary care and inpatients | 3296 | 699 | 21.2 | quantitative (VIDAS (bioMérieux) / Tinaquant (Roche)) |
| Goekoop RJ [23] | Thromb Haemost | 2007 | Referred secondary care | 876 | 110 | 12.6 | quantitative (VIDAS (bioMérieux)) |
| Runyon MS [24] | Emerg Med J | 2007 | Self-referral emergency care | 1187 | 39 | 3.3 | qualitative (NR) |
| Kline JA [25] | J Thromb Haemost | 2008 | Self-referral emergency care | 7889 | 563 | 7.1 | qualitative (NR) |
| Righini M [26] | Lancet | 2008 | Referred secondary care | 1692 | 361 | 21.3 | quantitative (VIDAS (bioMérieux)) |
| Douma RA [27] | Ann Itern Med | 2011 | Referred secondary care and inpatients | 807 | 192 | 23.8 | quantitative (VIDAS / Tinaquant / STA Liatest / Innovance) |
| Galipienzo J [28] | Rom J Intern Med | 2012 | Referred secondary care | 240 | 63 | 26.3 | quantitative (VIDAS (bioMérieux)) |
| Geersing GJ [1] | BMJ | 2012 | Primary healthcare | 597 | 73 | 12.2 | qualitative (Simplify) |
| Kline JA [29] | J Thromb Haemost | 2012 | Self-referral emergency care and inpatients | 678 | 115 | 17.0 | quantitative (VIDAS (bioMérieux)) |
| Mos ICM [30] | Thromb Res | 2014 | Referred secondary care and inpatients | 279 | 114 | 40.9 | quantitative (Tinaqaunt / VIDAS / STA Liatest / Innovance) |
| Righini M [31] | JAMA | 2014 | Referred secondary care | 3324 | 639 | 19.2 | quantitative (VIDAS / Tinaquant / Cobas h 232 / SA Liatest / HS-500 / Innovance) |
| Schouten HJ [32] | J Am Geriatr Soc | 2014 | Primary healthcare and nursing homes | 129 | 51 | 39.8 | quantitative (NR) |
| Penaloza A [33] | Lancet Haematol | 2017 | Referred secondary care | 705 | 153 | 21.7 | quantitative (NR) |
| van der Hulle T [34] | Lancet | 2017 | Referred secondary care and inpatients | 3448 | 473 | 13.7 | quantitative (VIDAS / Tinaquant / STA Liatest / Innovance) |
| Kearon C [35] | N Engl J Med | 2019 | Primary healthcare and inpatients | 2017 | 150 | 7.4 | quantitative (STA Liatest / HS 500 / Innovance / Triage / Other) |

^a^ These values are based on the data provided by the original authors.

Abbreviations: PE, pulmonary embolism; NR, not reported

**References**

1. Geersing GJ, Erkens PM, Lucassen WA, Buller HR, Cate HT, Hoes AW, et al. Safe exclusion of pulmonary embolism using the Wells rule and qualitative D-dimer testing in primary care: prospective cohort study. BMJ. 2012;345:e6564. Epub 2012/10/06. doi: 10.1136/bmj.e6564. PubMed PMID: 23036917; PubMed Central PMCID: PMC3464185.

2. Kearon C, Ginsberg JS, Douketis J, Turpie AG, Bates SM, Lee AY, et al. An evaluation of D-dimer in the diagnosis of pulmonary embolism: a randomized trial. Ann Intern Med. 2006;144(11):812-21. Epub 2006/06/07. doi: 10.7326/0003-4819-144-11-200606060-00007. PubMed PMID: 16754923.

3. Sanson BJ, Lijmer JG, Mac Gillavry MR, Turkstra F, Prins MH, Buller HR. Comparison of a clinical probability estimate and two clinical models in patients with suspected pulmonary embolism. ANTELOPE-Study Group. Thromb Haemost. 2000;83(2):199-203. Epub 2000/03/30. PubMed PMID: 10739372.

4. Janssen KJ, Donders AR, Harrell FE, Jr., Vergouwe Y, Chen Q, Grobbee DE, et al. Missing covariate data in medical research: to impute is better than to ignore. J Clin Epidemiol. 2010;63(7):721-7. Epub 2010/03/27. doi: 10.1016/j.jclinepi.2009.12.008. PubMed PMID: 20338724.

5. Donders AR, van der Heijden GJ, Stijnen T, Moons KG. Review: a gentle introduction to imputation of missing values. J Clin Epidemiol. 2006;59(10):1087-91. Epub 2006/09/19. doi: 10.1016/j.jclinepi.2006.01.014. PubMed PMID: 16980149.

6. Geersing GJ, Kraaijpoel N, Buller HR, van Doorn S, van Es N, Le Gal G, et al. Ruling out pulmonary embolism across different subgroups of patients and healthcare settings: protocol for a systematic review and individual patient data meta-analysis (IPDMA). Diagn Progn Res. 2018;2:10. Epub 2019/05/17. doi: 10.1186/s41512-018-0032-7. PubMed PMID: 31093560; PubMed Central PMCID: PMC6460525.

7. Audigier V, White IR, Jolani S, Debray TPA, Quartagno M, Carpenter J, et al. Multiple imputation for multilevel data with continuous and binary variables. Stat Sci. 33(2):160-83.

8. Quartagno M, Carpenter JR. Multiple imputation for IPD meta-analysis: allowing for heterogeneity and studies with missing covariates. Stat Med. 2016;35(17):2938-54. Epub 2015/12/19. doi: 10.1002/sim.6837. PubMed PMID: 26681666; PubMed Central PMCID: PMC5064632.

9. Quartagno M, Carpenter JR. Multiple imputation for discrete data: Evaluation of the joint latent normal model. Biom J. 2019;61(4):1003-19. Epub 2019/03/15. doi: 10.1002/bimj.201800222. PubMed PMID: 30868652; PubMed Central PMCID: PMC6618333.

10. Resche-Rigon M, White IR. Multiple imputation by chained equations for systematically and sporadically missing multilevel data. Stat Methods Med Res. 2018;27(6):1634-49. Epub 2016/09/21. doi: 10.1177/0962280216666564. PubMed PMID: 27647809; PubMed Central PMCID: PMC5496677.

11. Geersing GJ, Zuithoff NP, Kearon C, Anderson DR, Ten Cate-Hoek AJ, Elf JL, et al. Exclusion of deep vein thrombosis using the Wells rule in clinically important subgroups: individual patient data meta-analysis. BMJ. 2014;348:g1340. Epub 2014/03/13. doi: 10.1136/bmj.g1340. PubMed PMID: 24615063; PubMed Central PMCID: PMC3948465.

12. Kline JA, Nelson RD, Jackson RE, Courtney DM. Criteria for the safe use of D-dimer testing in emergency department patients with suspected pulmonary embolism: a multicenter US study. Ann Emerg Med. 2002;39(2):144-52. Epub 2002/02/02. doi: 10.1067/mem.2002.121398. PubMed PMID: 11823768.

13. Kline JA, Runyon MS, Webb WB, Jones AE, Mitchell AM. Prospective study of the diagnostic accuracy of the simplify D-dimer assay for pulmonary embolism in emergency department patients. Chest. 2006;129(6):1417-23. Epub 2006/06/17. doi: 10.1378/chest.129.6.1417. PubMed PMID: 16778257.

14. Riley RD, Dodd SR, Craig JV, Thompson JR, Williamson PR. Meta-analysis of diagnostic test studies using individual patient data and aggregate data. Stat Med. 2008;27(29):6111-36. Epub 2008/09/26. doi: 10.1002/sim.3441. PubMed PMID: 18816508.

15. Simel DL, Bossuyt PM. Differences between univariate and bivariate models for summarizing diagnostic accuracy may not be large. J Clin Epidemiol. 2009;62(12):1292-300. Epub 2009/05/19. doi: 10.1016/j.jclinepi.2009.02.007. PubMed PMID: 19447007.

16. Dronkers CEA, van der Hulle T, Le Gal G, Kyrle PA, Huisman MV, Cannegieter SC, et al. Towards a tailored diagnostic standard for future diagnostic studies in pulmonary embolism: communication from the SSC of the ISTH. J Thromb Haemost. 2017;15(5):1040-3. Epub 2017/03/16. doi: 10.1111/jth.13654. PubMed PMID: 28296048.

17. Lucassen W, Geersing GJ, Erkens PM, Reitsma JB, Moons KG, Buller H, et al. Clinical decision rules for excluding pulmonary embolism: a meta-analysis. Ann Intern Med. 2011;155(7):448-60. Epub 2011/10/05. doi: 10.7326/0003-4819-155-7-201110040-00007. PubMed PMID: 21969343.

18. Wicki J, Perneger TV, Junod AF, Bounameaux H, Perrier A. Assessing clinical probability of pulmonary embolism in the emergency ward: a simple score. Arch Intern Med. 2001;161(1):92-7. Epub 2001/01/09. doi: 10.1001/archinte.161.1.92. PubMed PMID: 11146703.

19. Perrier A, Roy PM, Aujesky D, Chagnon I, Howarth N, Gourdier AL, et al. Diagnosing pulmonary embolism in outpatients with clinical assessment, D-dimer measurement, venous ultrasound, and helical computed tomography: a multicenter management study. Am J Med. 2004;116(5):291-9. Epub 2004/02/27. doi: 10.1016/j.amjmed.2003.09.041. PubMed PMID: 14984813.

20. Ghanima W, Almaas V, Aballi S, Dorje C, Nielssen BE, Holmen LO, et al. Management of suspected pulmonary embolism (PE) by D-dimer and multi-slice computed tomography in outpatients: an outcome study. J Thromb Haemost. 2005;3(9):1926-32. Epub 2005/08/17. doi: 10.1111/j.1538-7836.2005.01544.x. PubMed PMID: 16102097.

21. Perrier A, Roy PM, Sanchez O, Le Gal G, Meyer G, Gourdier AL, et al. Multidetector-row computed tomography in suspected pulmonary embolism. N Engl J Med. 2005;352(17):1760-8. Epub 2005/04/29. doi: 10.1056/NEJMoa042905. PubMed PMID: 15858185.

22. van Belle A, Buller HR, Huisman MV, Huisman PM, Kaasjager K, Kamphuisen PW, et al. Effectiveness of managing suspected pulmonary embolism using an algorithm combining clinical probability, D-dimer testing, and computed tomography. JAMA. 2006;295(2):172-9. Epub 2006/01/13. doi: 10.1001/jama.295.2.172. PubMed PMID: 16403929.

23. Goekoop RJ, Steeghs N, Niessen RW, Jonkers GJ, Dik H, Castel A, et al. Simple and safe exclusion of pulmonary embolism in outpatients using quantitative D-dimer and Wells' simplified decision rule. Thromb Haemost. 2007;97(1):146-50. Epub 2007/01/04. PubMed PMID: 17200782.

24. Runyon MS, Beam DM, King MC, Lipford EH, Kline JA. Comparison of the Simplify D-dimer assay performed at the bedside with a laboratory-based quantitative D-dimer assay for the diagnosis of pulmonary embolism in a low prevalence emergency department population. Emerg Med J. 2008;25(2):70-5. Epub 2008/01/24. doi: 10.1136/emj.2007.048918. PubMed PMID: 18212136.

25. Kline JA, Courtney DM, Kabrhel C, Moore CL, Smithline HA, Plewa MC, et al. Prospective multicenter evaluation of the pulmonary embolism rule-out criteria. J Thromb Haemost. 2008;6(5):772-80. Epub 2008/03/06. doi: 10.1111/j.1538-7836.2008.02944.x. PubMed PMID: 18318689.

26. Righini M, Le Gal G, Aujesky D, Roy P-M, Sanchez O, Verschuren F, et al. Diagnosis of pulmonary embolism by multidetector CT alone or combined with venous ultrasonography of the leg: a randomised non-inferiority trial. Lancet. 2008;371(9621):1343-52. doi: 10.1016/s0140-6736(08)60594-2.

27. Douma RA, Mos IC, Erkens PM, Nizet TA, Durian MF, Hovens MM, et al. Performance of 4 clinical decision rules in the diagnostic management of acute pulmonary embolism: a prospective cohort study. Ann Intern Med. 2011;154(11):709-18. Epub 2011/06/08. doi: 10.7326/0003-4819-154-11-201106070-00002. PubMed PMID: 21646554.

28. Galipienzo J, Garcia de Tena J, Flores J, Alvarez C, Garcia-Avello A, Arribas I. Effectiveness of a diagnostic algorithm combining clinical probability, D-dimer testing, and computed tomography in patients with suspected pulmonary embolism in an emergency department. Rom J Intern Med. 2012;50(3):195-202. Epub 2013/01/22. PubMed PMID: 23330286.

29. Kline JA, Hogg MM, Courtney DM, Miller CD, Jones AE, Smithline HA. D-dimer threshold increase with pretest probability unlikely for pulmonary embolism to decrease unnecessary computerized tomographic pulmonary angiography. J Thromb Haemost. 2012;10(4):572-81. doi: 10.1111/j.1538-7836.2012.04647.x.

30. Mos ICM, Douma RA, Erkens PMG, Kruip MJHA, Hovens MM, van Houten AA, et al. Diagnostic outcome management study in patients with clinically suspected recurrent acute pulmonary embolism with a structured algorithm. Thromb Res. 2014;133(6):1039-44. doi: 10.1016/j.thromres.2014.03.050.

31. Righini M, Van Es J, Den Exter PL, Roy PM, Verschuren F, Ghuysen A, et al. Age-adjusted D-dimer cutoff levels to rule out pulmonary embolism: the ADJUST-PE study. JAMA. 2014;311(11):1117-24. Epub 2014/03/20. doi: 10.1001/jama.2014.2135. PubMed PMID: 24643601.

32. Schouten HJ, Geersing GJ, Oudega R, van Delden JJ, Moons KG, Koek HL. Accuracy of the Wells clinical prediction rule for pulmonary embolism in older ambulatory adults. J Am Geriatr Soc. 2014;62(11):2136-41. Epub 2014/11/05. doi: 10.1111/jgs.13080. PubMed PMID: 25366538.

33. Penaloza A, Soulié C, Moumneh T, Delmez Q, Ghuysen A, El Kouri D, et al. Pulmonary embolism rule-out criteria (PERC) rule in European patients with low implicit clinical probability (PERCEPIC): a multicentre, prospective, observational study. Lancet Haematol. 2017;4(12):e615-e21. doi: 10.1016/s2352-3026(17)30210-7.

34. van der Hulle T, Cheung WY, Kooij S, Beenen LFM, van Bemmel T, van Es J, et al. Simplified diagnostic management of suspected pulmonary embolism (the YEARS study): a prospective, multicentre, cohort study. Lancet. 2017;390(10091):289-97. doi: 10.1016/s0140-6736(17)30885-1.

35. Kearon C, de Wit K, Parpia S, Schulman S, Afilalo M, Hirsch A, et al. Diagnosis of Pulmonary Embolism with d-Dimer Adjusted to Clinical Probability. N Engl J Med. 2019;381(22):2125-34. doi: 10.1056/NEJMoa1909159.
