# Supplementary material for: Ruling out pulmonary embolism across different healthcare settings: A systematic review and individual patient data meta-analysis
Source: PLoS Med. 2022 Jan 25;19(1):e1003905. doi: 10.1371/journal.pmed.1003905 (PMC8824365; doi:10.1371/journal.pmed.1003905)
Supplement: S1 Fig — Includes Fig A Flow of studies, Fig B Risk of bias assessment, Fig C The range of failure rate and efficiency of the diagnostic strategies with I2 statistics, Fig D Sensitivity analysis including only studies in which all diagnostic strategies can be calculated, and Fig E Sensitivity analysis including only studies in which all diagnostic strategies except PERC algorithm can be calculated. PERC, Pulmonary Embolism Rule-out Criteria. (DOCX) [file pmed.1003905.s003.docx]

**S1 Figs**


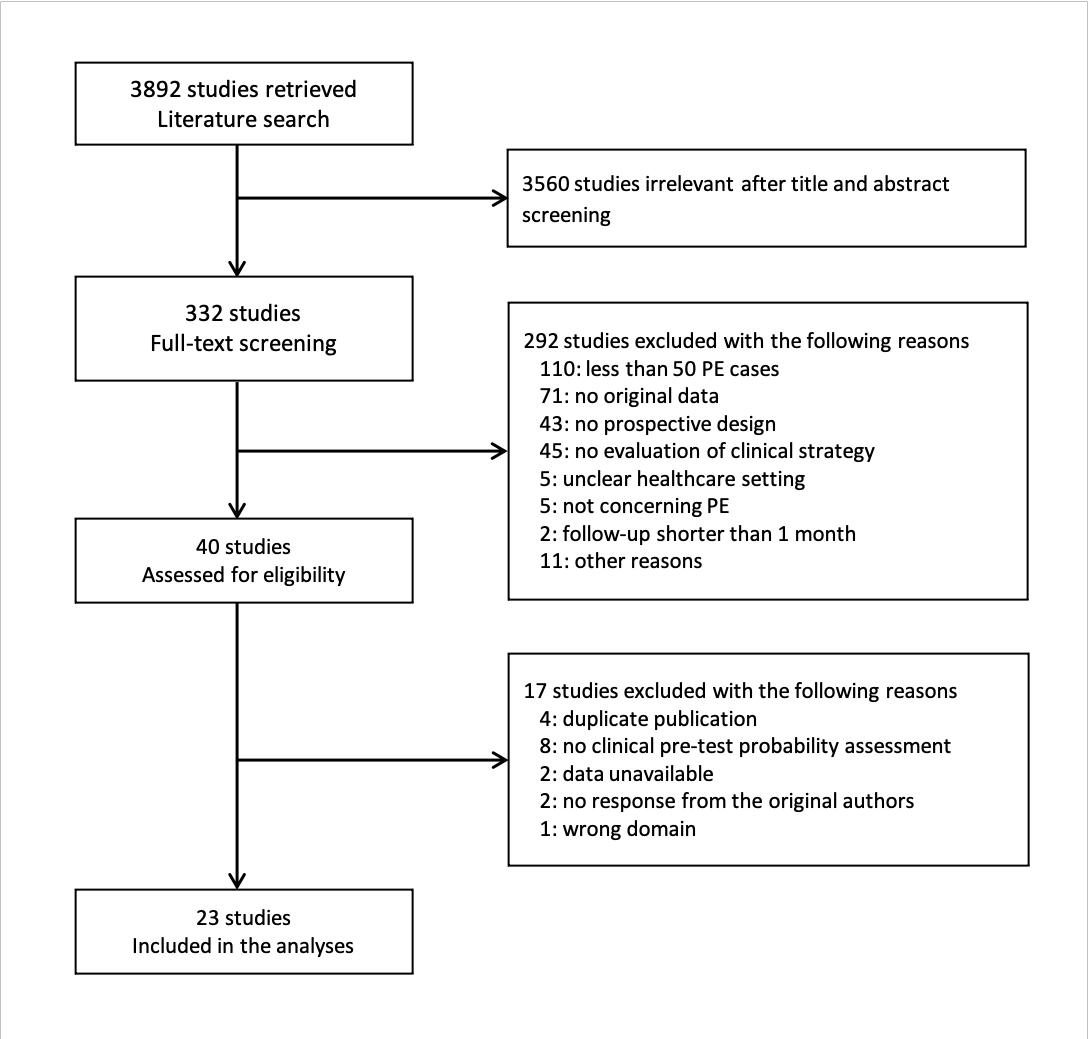
**Figure A: Flow of studies**

Abbreviations: PE, pulmonary embolism

**
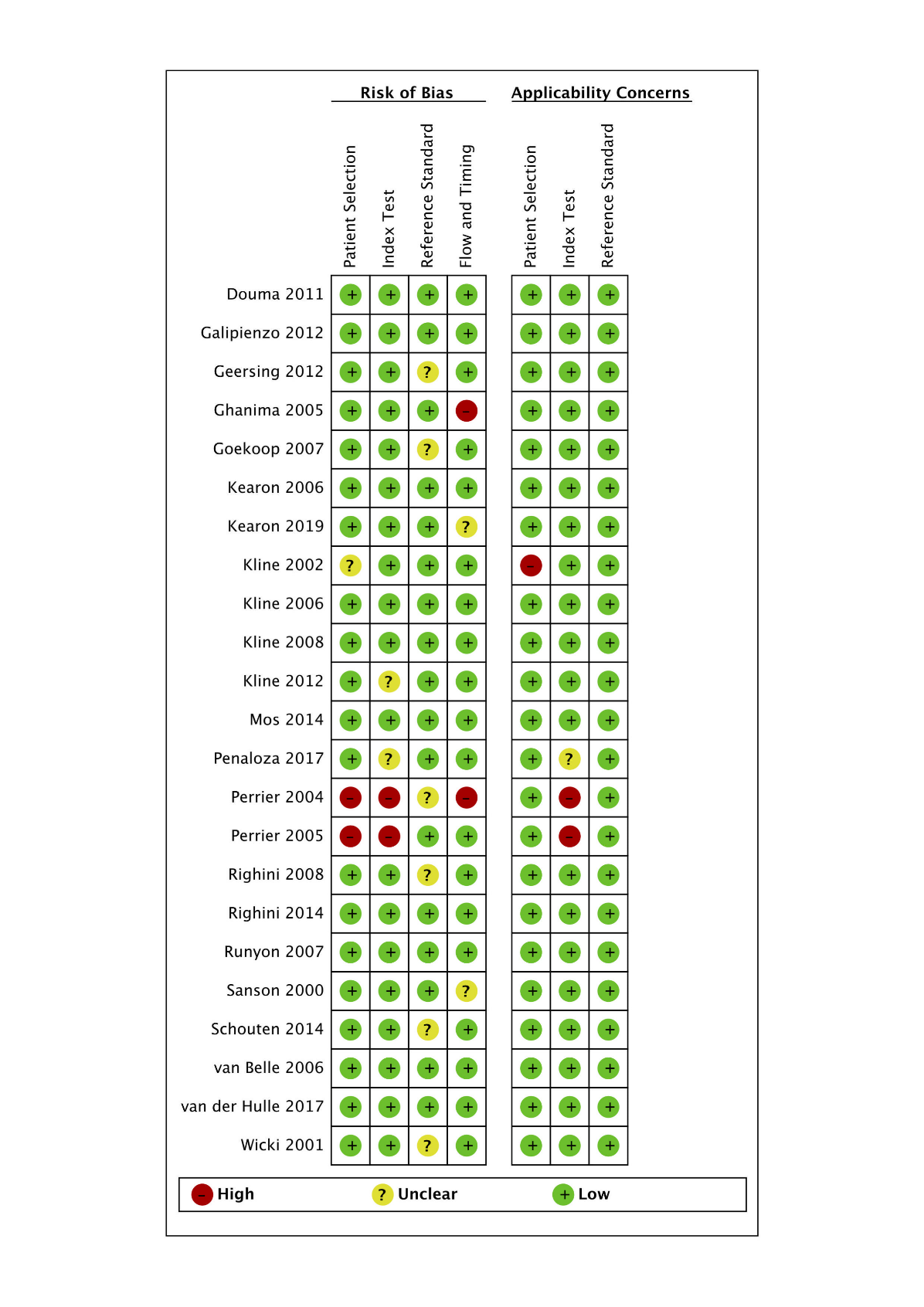
Figure B: Risk of bias assessment**

**
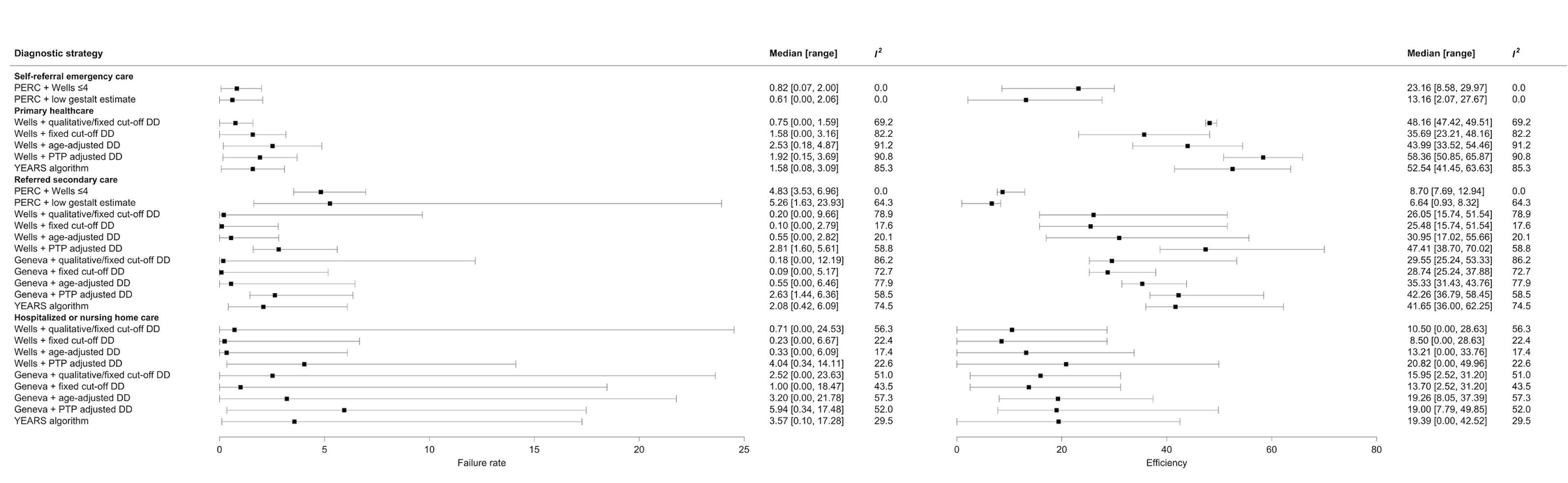
Figure C: The range of failure rate and efficiency of the diagnostic strategies with *I^2^* statistics**

**
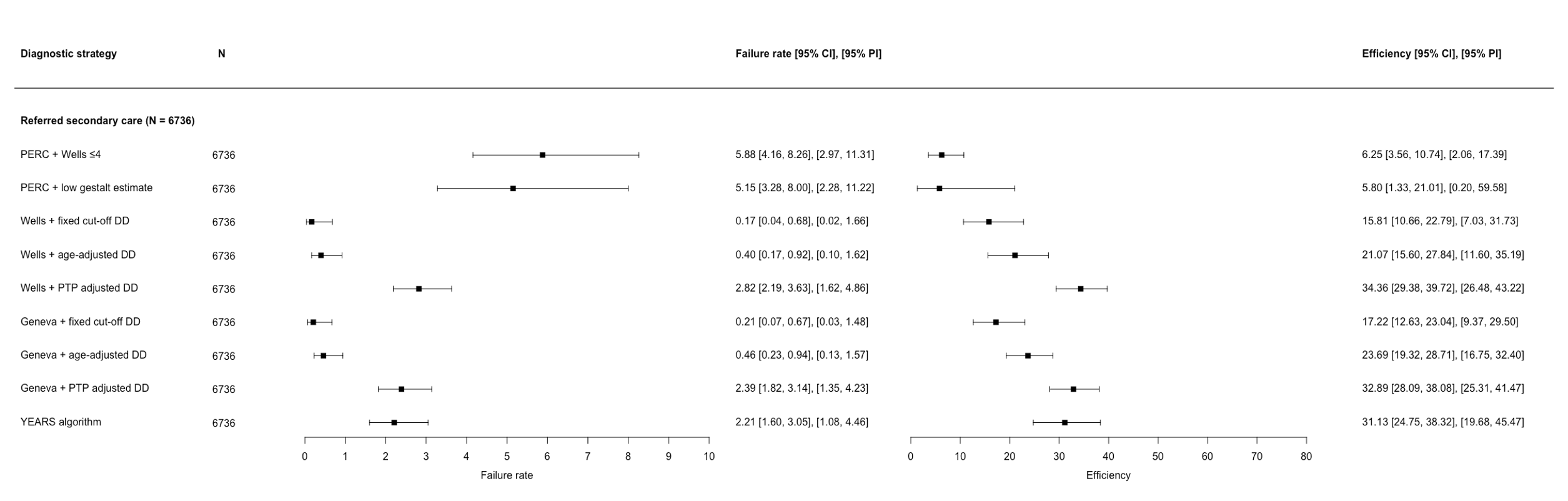
Figure D: Sensitivity analysis including only studies in which all diagnostic strategies can be calculated**

Abbreviations: N, number of patients; CI, confidence interval; PI, prediction interval; PTP, pre-test probability; DD, D-dimer

**Figure E: Sensitivity analysis including only studies in which all diagnostic strategies except PERC algorithm can be calculated**

**
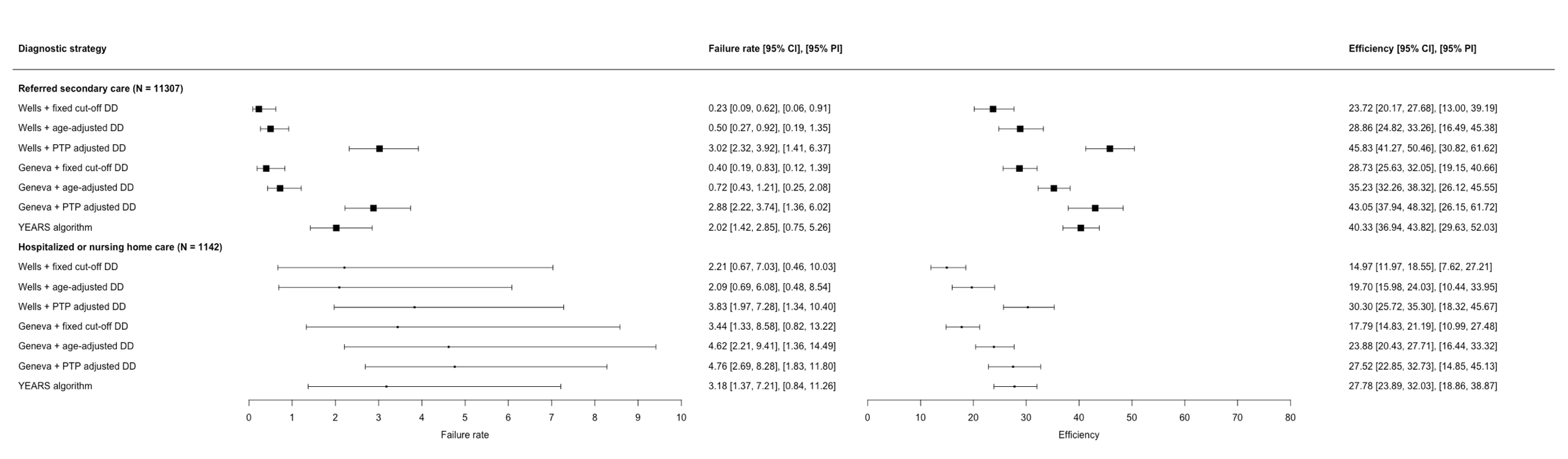
**

Abbreviations: N, number of patients; CI, confidence interval; PI, prediction interval; PTP, pre-test probability; DD, D-dimer
