# Supplementary material for: Ruling out pulmonary embolism across different healthcare settings: A systematic review and individual patient data meta-analysis
Source: PLoS Med. 2022 Jan 25;19(1):e1003905. doi: 10.1371/journal.pmed.1003905 (PMC8824365; doi:10.1371/journal.pmed.1003905)
Supplement: Attachment — Submitted filename: Rebuttal_R2.docx [file pmed.1003905.s004.docx]

Dear Editors,

Please find enclosed the revised manuscript entitled: “Ruling-out pulmonary embolism across different healthcare settings: A systematic review and individual patient data meta-analysis”

We very much appreciated all the additional comments and kind suggestions of the editors and the reviewer, and we have revised our manuscript accordingly. Please find below our detailed response to their comments.

We hope you will find our revised manuscript suitable for publication in *PLOS Medicine*, yet should any questions remain, we are more than happy to respond to those as well.

Yours sincerely,

Of behalf of our co-authors,

Geert-Jan Geersing, MD, PhD

Toshihiko Takada, MD, PhD

Julius Center for Health Sciences and Primary Care, University Medical Center Utrecht, Utrecht University, Universiteitsweg 100, 3584 CG Utrecht, The Netherlands

**Requests from Editors:**

Lines 76-80: Please update to ‘The main limitation of the primary analyses was that the diagnostic performance of each strategy was compared in different sets of studies since the availability of items used in each diagnostic strategy differed across included studies, however sensitivity analyses suggest the findings were robust’, or similar.

***Response:*** We have revised the sentence accordingly. (page 4, line 76-79)

Line 139: ‘The secondary aim’

***Response:*** We have revised it accordingly. (page 7, line 138)

The titles of Figure S3/S4 appear to be missing some text.

***Response:*** We thank the editors for careful reading throughout the manuscript. We have revised the title of the figures as follows (Note: Figure numbers have been changed as we have added a new supplementary figure, prompted by the reviewers’ comment on I2’s, see below):

S4 Figure: Sensitivity analysis including only studies in which all diagnostic strategies can be calculated (page 33, line 630-631)

S5 Figure: Sensitivity analysis including only studies in which all diagnostic strategies except PERC algorithm can be calculated (page 33, line 632-633)

Line 379: ‘Considerably’

***Response:*** We have revised it accordingly. (page 22, line 380)

Please remove the Strengths and Limitations subheading in your Discussion.

***Response:*** We have removed the subheading.

Please remove the Acknowledgements and Data availability sections (the former having no content and the latter being captured in your submission form response).

***Response:*** Those sections have been removed from the manuscript.

**Comments from Reviewers:**

Reviewer #1: I thank the authors for the detailed response to my comments and comments of the editor and other reviewers. I think the revised version looks good. I just have two minor additional comments.

***Response:*** We thank the reviewer for their thorough review and these kind words.

1. Line 232-233: please make sure to include this template in the appendix. Maybe I did not look carefully enough but I did not find this template in the main text or in the appendix.

***Response:*** We agree with the reviewer that the template should be attached in the manuscript. Now, it has been added as S2 Table.

2. Line 258-260: I see that you are trying to use a new approach to examine the between study heterogeneity. Although I think you justify the method you used, I still believe it would be helpful to also provide the I squared statistics since most people are familiar with that statistics and it's a custom to provide that in a meta-analysis.

***Response:*** Prompted by the reviewer’s comment, we have now added supplementary figures to show the range of failure rate and efficiency of each diagnostic strategy in included studies and *I^2^* to visualize between-study heterogeneity, in addition to our reported 95% prediction intervals.

In Methods section:

In addition, the range of failure rate and efficiency of each diagnostic strategy in included studies was visualized with *I^2^* [25]. (page 12, line 261-262)

In Results section:

The range of failure rate and efficiency in the included studies are shown with *I^2^* in S3 Figure. (page 16, line 302-303)

After these two issues are checked/fixed, I think this paper is ready for publication. Congratulations!

***Response:*** We thank the reviewer for this kind word. We hope our revised manuscript is now suitable for publication in *PLOS Medicine*.
